# Acute Injection of Omega-3 Triglyceride Emulsion Provides Very Similar Protection as Hypothermia in a Neonatal Mouse Model of Hypoxic-Ischemic Brain Injury

**DOI:** 10.3389/fneur.2020.618419

**Published:** 2021-01-15

**Authors:** Denny Joseph Manual Kollareth, Hylde Zirpoli, Vadim S. Ten, Richard J. Deckelbaum

**Affiliations:** ^1^Institute of Human Nutrition, Columbia University Irving Medical Center, New York, NY, United States; ^2^Department of Pediatrics, Vagelos College of Physicians and Surgeons, Columbia University Irving Medical Center, New York, NY, United States

**Keywords:** DHA, hypothermia, hypoxic-ischemic injury, neuroprotection, omega-3 fatty acids, stroke

## Abstract

Therapeutic hypothermia (HT) is a currently accepted treatment for neonatal asphyxia and is a promising strategy in adult stroke therapy. We previously reported that acute administration of docosahexaenoic acid (DHA) triglyceride emulsion (tri-DHA) protects against hypoxic-ischemic (HI) injury in neonatal mice. We questioned if co-treatment with HT and tri-DHA would achieve synergic effects in protecting the brain from HI injury. Neonatal mice (10-day old) subjected to HI injury were placed in temperature-controlled chambers for 4 h of either HT (rectal temperature 31–32°C) or normothermia (NT, rectal temperature 37°C). Mice were treated with tri-DHA (0.375 g tri-DHA/kg bw, two injections) before and 1 h after initiation of HT. We observed that HT, beginning immediately after HI injury, reduced brain infarct volume similarly to tri-DHA treatment (~50%). Further, HT delayed 2 h post-HI injury provided neuroprotection (% infarct volume: 31.4 ± 4.1 vs. 18.8 ± 4.6 HT), while 4 h delayed HT did not protect against HI insult (% infarct volume: 30.7 ± 5.0 vs. 31.3 ± 5.6 HT). HT plus tri-DHA combination treatment beginning at 0 or 2 h after HI injury did not further reduce infarct volumes compared to HT alone. Our results indicate that HT offers similar degrees of neuroprotection against HI injury compared to tri-DHA treatment. HT can only be provided in tertiary care centers, requires intense monitoring and can have adverse effects. In contrast, tri-DHA treatment may be advantageous in providing a feasible and effective strategy in patients after HI injury.

## Introduction

Hypoxic-ischemic (HI) brain injury is a serious occurrence that frequently results in death or significant long-term neurologic disability in both neonates and adults (1–3). Currently, therapeutic hypothermia (HT) is the only established treatment for neonates with HI encephalopathy (4). Selective head cooling with cooling caps or whole body cooling with passive cooling (turning radiant warmers/incubators off), cool packs and/or commercially available cooling blankets are used for treatment in neonatal HI encephalopathy (5, 6). With regard to acute ischemic stroke in adults, tissue-type plasminogen activator (tPA) is the only drug approved by the U.S. Food and Drug Administration (FDA) (7). However, the narrow therapeutic window and the risk of hemorrhage are major limitations of tPA treatment, resulting in only 8–10% of adult stroke patients eligible for this drug (8). Preclinical studies and small scale clinical trials in adults after stroke have shown that HT substantially diminishes the degree of neural damage, reduces the rate of mortality and improves neurofunctional recovery (9–11).

The major molecular mechanisms affected by HT include decreased free-radical production, reduction of blood–brain barrier disruption, decreased excitatory amino acid release and attenuation of cell mediated inflammatory responses to cerebral ischemia (12, 13). Additionally, HT induces the inhibition of neuronal apoptosis through both mitochondrial based intrinsic pathways and receptor mediated extrinsic pathways (14). However, HT remains a complex medical approach, as it requires intense monitoring and is available only in tertiary care centers (15). Pilot studies on HT in stroke have shown that adult patients have less tolerance to cooling than neonates and HT may also induce unfavorable systemic effects, such as shivering, immune suppression, and pneumonia (16, 17). Combining HT with other treatment methods may help in reducing the adverse effects from HT as well as reaching multiple molecular targets in the setting of HI insult to obtain an increase in therapeutic time windows and an enhanced repair in long-term recovery (18).

As one of the major omega-3 polyunsaturated fatty acids (PUFA) in the brain, docosahexaenoic acid (DHA) is essential for development and function of the brain (19). DHA has been shown to reduce inflammation, excitotoxicity and to prevent brain volume loss in different animal models of HI injury (20–22). Studies from our laboratory showed that acute administration of triglyceride (TG) emulsions containing only DHA (tri-DHA) reduces brain injury and preserves short- and long-term neurological outcomes in neonatal mice (23, 24).

Based on these findings, we questioned if co-treatment with HT and tri-DHA would achieve synergic effects in protecting the brain from HI injury. We validated the neuroprotective efficacy of HT against HI injury in the neonatal model previously described by our laboratory (23, 25). Our results showed that tri-DHA provides similar degrees of neuroprotection as that of HT and combining HT with tri-DHA emulsion does not offer additional therapeutic benefit in HI injury.

## Materials and Methods

### Ethics Statement

All research studies were carried out according to protocols approved by the Columbia University Institutional Animal Care and Use Committee (IACUC) in accordance with the Association for Assessment and Accreditation of Laboratory Animal Care guidelines (AAALAC).

### Materials

DHA TG oil was purchased from Nu-Chek Prep, Inc. (Elysian, MN). Egg yolk phosphatidylcholine was obtained from Avanti Polar-Lipids, Inc. (Alabaster, AL). Radiolabeled [^3^H]-cholesteryl hexadecyl ether was purchased from PerkinElmer (Boston, MA) ([^3^H]CEt) (NET 85900).

### Lipid Emulsions

Tri-DHA emulsions (10 g by TG weight/100 mL emulsion) were made in our laboratory with DHA TG oil and egg yolk phospholipids (PL) by sonication as previously detailed (23). The emulsions were analyzed for the amount of TG and PL using commercial kits (Wako Chemicals USA, Inc., Richmond, VA). The TG:PL mass ratio was 5.0 ± 1.0, similar to VLDL-sized particles. To prepare radiolabeled emulsions, [^3^H]CEt was added to the TG-PL mixture before sonication (25).

### Animal Procedures

#### Unilateral Cerebral Hypoxia-Ischemia Injury

Three-day-old C57BL/6J neonatal mice were purchased from Jackson Laboratories (Bar Harbor) with their birth mother. We used the Rice-Vannuci method of mild HI brain injury modified to 10-day old (p10) mice, as previously described (23). An initial pilot study on gender differences showed no significant changes in infarct volumes after HI injury between male and female mice. Hence, both male and female mice were used for these experiments and we did not separate our data by gender in the present study. Briefly, HI brain injury was induced by permanent ligation of the right common carotid artery. After 1.5 h of recovery, mice were exposed to hypoxic insult (humidified 8% O_2_/92% N_2_, Tech Air Inc., NY) for 15 min. Since HI brain injury in neonatal mice is associated with an endogenous drop in body core temperature (26), mice are kept at 37 ± 0.3°C during hypoxia to avoid hypothermia during the hypoxia period.

#### HT and Tri-DHA Treatments

Immediately after HI injury, pups were kept for 4 h in temperature controlled chambers with either HT or normothermia (NT), reaching rectal temperatures of 31–32°C or 37°C, respectively (23). We observed that pups placed in circulating air chambers set at 27°C maintained target rectal temperature 31–32°C. For the NT group, pups were placed in chambers set at 32°C, based on the protocol from our previous studies (23, 24). As the core temperature in neonatal rodents could be affected by distance from the dam (27), the pups were kept separately from the dam during the 4 h HT or NT treatment period. Sequential temperature measurements were obtained immediately after hypoxia (0 h) followed by 1, 2, 3, and 4 h during HT (probe type: RET-4; Physitemp Instruments, Clifton, NJ). Tri-DHA treatment [0.375 g tri-DHA/kg bw, intraperitoneal (i.p.), two injections, 1 h apart] was based on the protocol from our previous studies on tri-DHA neuroprotection against HI injury in neonatal mice (23, 24).

To investigate whether combined treatment of HT with tri-DHA emulsion enhances neuroprotection in HI damage, animals subjected to HT were administered with tri-DHA emulsion (0.375 g tri-DHA/kg bw, 2 injections, i.p.) at the beginning of HT and at 1 h after initiation of HT. NT or HT control animals received saline injections. Following 4 h NT, pups in the control group were returned to the dam. Pups in the HT group underwent slow rewarming by increasing the chamber temperature at a rate of 0.1–0.2°C per minute till the pups reached a rectal temperature of 37°C, and were then returned to the dam.

#### Uptake and Distribution of Radiolabeled Tri-DHA Emulsion Particles in HT Mice

Using radiolabeled tri-DHA emulsion, we determined whether HT affects the absorption and distribution of emulsion particles after i.p. injection. Naïve neonatal mice injected with radiolabeled tri-DHA emulsion (0.375 g tri-DHA/kg bw, i.p., single injection) were immediately subjected to 4 h of either HT (*n* = 3) or NT (*n* = 7). The use of a single bolus injection to study emulsion distribution was based on previously established protocols from our laboratory (25, 28). Animals were sacrificed after 4 h of HT or NT and radioactivity in peritoneal fluid, blood, organs and tissues assessed by measuring the levels of [^3^H]CEt.

Tissues and organs were homogenized using a Polytron Tissue Disruptor (Omni TH, Kenneswa, GA) and the radioactivity measured by liquid scintillation spectrometry (29). The samples were suspended in scintillation fluid (Ultima Gold scintillation fluid, PerkinElmer, Boston, MA), mixed and ^3^H dpm assayed in a PerkinElmer Tri-Carb liquid scintillation spectrometer 5110 TR. Tissue uptake was expressed as percent of total recovered dose/organ for all the organs analyzed.

#### HT and Tri-DHA Therapeutic Time Windows

We determined the therapeutic window of HT after HI injury in mice: (1) 2 h delayed HT - pups placed with dam for 2 h after HI and then subjected to HT; (2) 4 h delayed HT - pups placed with dam for 4 h after HI and then subjected to HT. To investigate whether combined treatment of HT with tri-DHA emulsion prolongs the therapeutic window in HI injury, animals subjected to HT (2 or 4 h delayed after HI) were administered with tri-DHA emulsion (0.375 g tri-DHA/kg bw, 2 injections, i.p.) at the beginning of HT and at 1 h after initiation of HT. NT or HT control animals received saline injections. After the treatment period, pups in NT or HT groups were returned to the dam as described above.

#### Neuropathological Outcomes

At 24 h after HI insult, the animals were sacrificed and brains were harvested. Coronal slices of 1 mm were cut by using a brain slicer matrix. Slices were immersed in a PBS solution containing 2% triphenyltetrazolium chloride (TTC) at 37°C for 25 min. TTC is taken up into living mitochondria, which converts it to a red color. Unstained areas that appeared white were defined as infarct regions whereas viable regions appeared red. Using Adobe Photoshop and NIH Image J imaging applications, planar areas of infarction on serial sections were summed to obtain the volume (mm^3^) of infarcted tissue. Infarct areas were expressed as % of the total area of the ipsilateral hemisphere (24). In a separate cohort of mice treated with HT or HT plus tri-DHA immediately after HI, brain atrophy at 7 days after HI injury was detected by Nissl staining, as previously described. The entire brain was sectioned every 200 μm and the thickness of each coronal slice was 50 μm. Sections were then incubated in a solution of 0.1% cresyl violet (Sigma-Aldrich, St. Louis, MO, USA) for 7 min. After a quick rinse in H_2_O, slides were differentiated in 70% (v/v) ethanol with a few drops of acetic acid, followed by dehydration in graded ethanol and two changes of xylene. The sections were then mounted with Fisher Chemical™ Permount™ Mounting Media (30).

### Statistical Analyses

Values are mean ± SEM. One-way ANOVA followed by *post hoc* Newman-Keuls multiple comparison test was applied to evaluate differences among the groups.

## Results

### HT Does Not Affect Absorption or Organ Distribution of Tri-DHA Emulsion Particles

There was no mortality in animals subjected to NT or HT protocols. Table 1 summarizes results of sequential temperature measurements in HT animals. Radiolabeled experiments showed that at 4 h after i.p. injection, ~96% of the injected emulsion exited the peritoneal cavity in both NT and HT mice. Further, no significant differences were observed in the organ distribution of tri-DHA emulsion particles in NT vs. HT mice. The highest uptake of emulsion particles was in the liver (44–47% of recovered dose of radiolabeled emulsion), followed by muscle (20–23%) and heart (8–9%) in both NT and HT mice. The lowest uptake of emulsion particles was in the brain (<0.3% of recovered dose) in both NT and HT animals (data not shown).

**Table 1:** Rectal temperature measurements immediately after hypoxia (0 h) and at 1, 2, 3, and 4 h during hypothermia (HT) in mice subjected to hypoxic-ischemic (HI) injury.

	**HT**	**HT + tri-DHA**
End of hypoxia (0 h)	35.2 ± 0.48	35.6 ± 0.41
1 h after HI	32.0 ± 0.16	32.2 ± 0.25
2 h after HI	31.4 ± 0.24	32.0 ± 0.17
3 h after HI	31.0 ± 0.19	32.1 ± 0.15
4 h after HI	31.6 ± 0.26	31.8 ± 0.28

*Temperatures (°C) are expressed as mean ± SEM. n = 7–9*.

### HT or Tri-DHA Treatment After HI Injury Provides Similar Degrees of Neuroprotection

**Figure 1:**
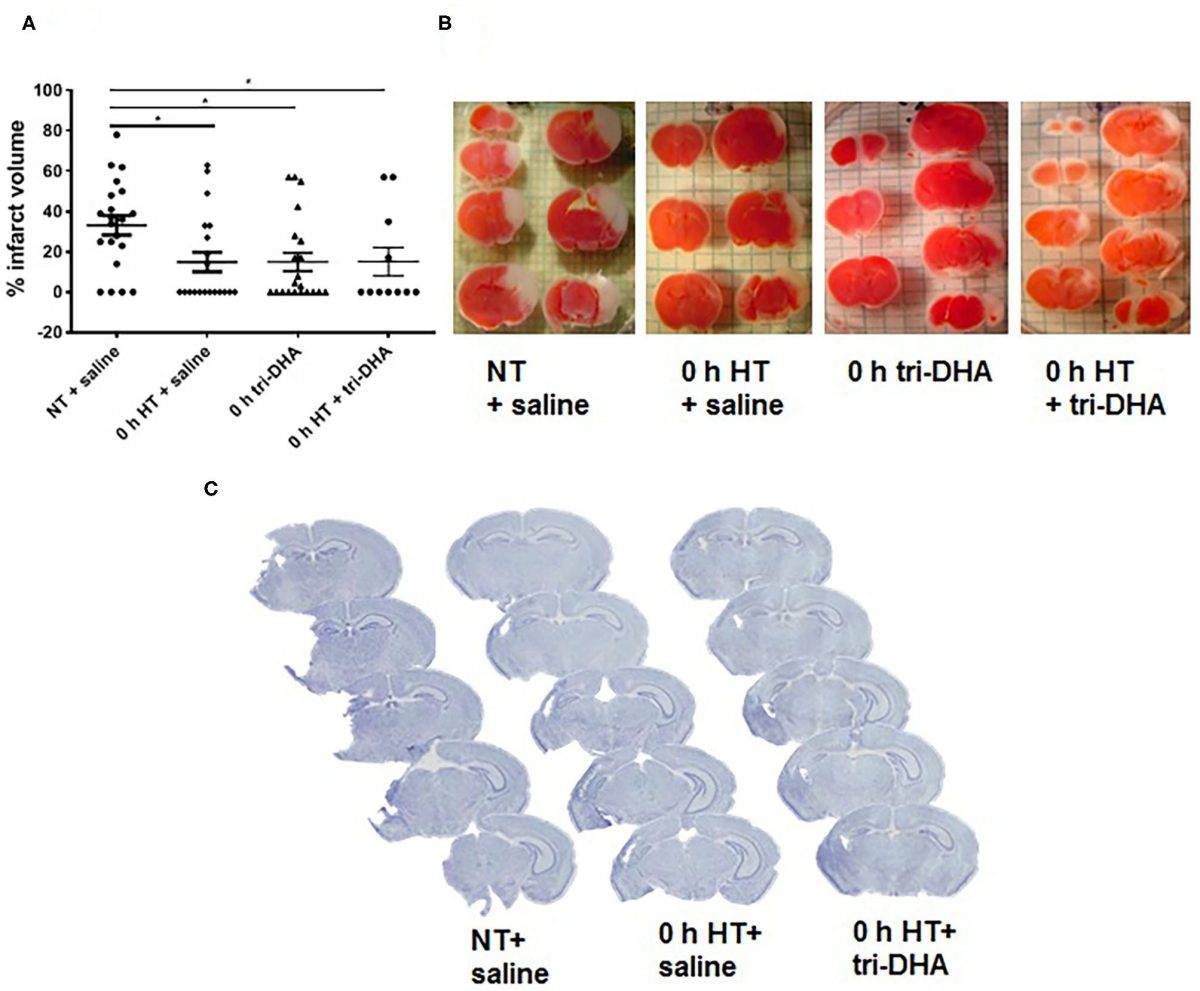
Effect of hypothermia (HT) and tri-DHA on infarct volume: Infarct volume **(A)** and representative TTC stained cerebral sections **(B)** in mice treated with normothermia (NT) + saline (*n* = 21), HT + saline (*n* = 20), tri-DHA (*n* = 21) or HT + tri-DHA (*n* = 11) beginning immediately (0 h) after hypoxic-ischemic (HI) injury (% infarct volume: NT + saline- 33.2 ± 4.8, HT + saline- 14.9 ± 4.8, 0 h tri-DHA- 14.9 ± 4.5, 0 h HT + tri-DHA- 15.0 ± 7.0). Values are mean ± SEM. *p < 0.05; Representative Nissl-stained cerebral coronal sections from mice treated with NT + saline, HT + saline or HT + tri-DHA beginning immediately (0 h) after HI injury **(C)**.

We evaluated neuroprotective effects of HT plus tri-DHA treatment beginning immediately after HI injury. HT or tri-DHA showed significant reduction (~50%) in brain infarct volumes compared to saline treated NT animals (Figures 1A,B). Combination of treatments with HT and tri-DHA immediately after HI injury did not provide any additional benefits compared to HT treatment alone (Figures 1A,B).

Neuroprotection by HT plus tri-DHA administration beginning immediately after HI injury was maintained at 7 days after ischemic insult. Nissl staining demonstrated greater preservation of the ipsilateral hemisphere in HT or HT plus tri-DHA treated mice compared to the control group. However, the combination did not offer any therapeutic advantage compared to HT treatment alone. Representative Nissl stained sections are shown in Figure 1C.

### HT Plus Tri-DHA Treatment After HI Injury Does Not Extend the Therapeutic Time Window

**Figure 2:**
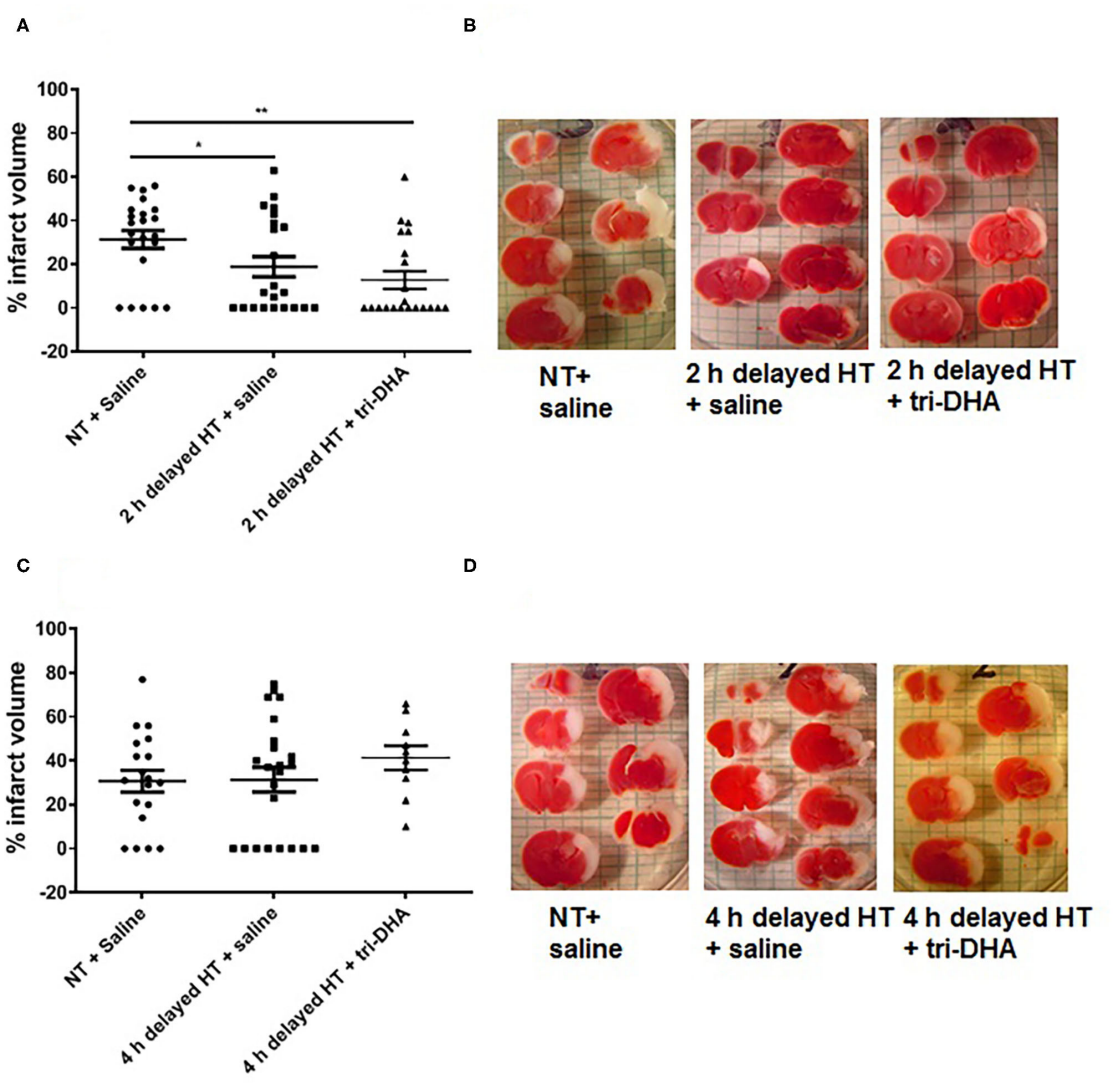
Therapeutic windows of hypothermia (HT) and tri-DHA: Infarct volume **(A)** and representative TTC stained cerebral sections **(B)** in mice treated with normothermia (NT) + saline (*n* = 22), HT + saline (*n* = 22) or HT + tri-DHA (*n* = 21) beginning at 2 h after hypoxic-ischemic (HI) injury (% infarct volume: NT + saline- 31.4 ± 4.1, 2 h delayed HT + saline- 18.8 ± 4.6, 2 h delayed HT + tri-DHA- 12.7 ± 4.0); Infarct volume **(C)** and representative TTC stained cerebral sections **(D)** in mice treated with normothermia (NT) + saline (*n* = 19), HT + saline (*n* = 23) or HT + tri-DHA (*n* = 10) beginning at 4 h after hypoxic-ischemic (HI) injury (% infarct volume: NT + saline- 30.7 ± 5.0, 4 h delayed HT + saline- 31.3 ± 5.6, 4 h delayed HT + tri-DHA- 41.3 ± 5.5). Values are mean ± SEM. *p < 0.05, **p < 0.01.

In the present study, we performed delayed HT treatment protocols to determine the therapeutic window for neuroprotection after ischemic injury. HT delayed 2 h post-HI showed reduced brain infarct volumes compared to NT animals. Further, HT plus tri-DHA treatment did not offer significant additional protection over that provided by HT alone beginning at 2 h after HI injury although there was a tendency for slightly more reduction in infarct size (% infarct volume: 31.4 ± 4.1 NT + saline vs. 18.8 ± 4.6 HT + saline vs. 12.7 ± 4.0 HT + tri-DHA) (Figures 2A,B). HT treatment delayed to 4 h after HI insult did not offer protection against ischemic injury. Combining HT and tri-DHA treatment with a delay of 4 h after HI injury did not extend the therapeutic window of HT. Although we observed an increase in infarct volume in animals treated with 4 h delayed HT + tri-DHA combination, the difference was not significant compared to NT or HT alone groups (Figures 2C,D). Thus, our results indicate that combined treatment of tri-DHA emulsion with HT does not provide additional significant benefit in neuroprotection in ischemic injury.

## Discussion

In this study, our results show that HT administration exerts similar degrees of neuroprotection as that of tri-DHA. Further, combined treatment of HT with tri-DHA emulsion does not confer additional neuroprotection.

Therapeutic HT is a means of neuroprotection well established in the management of acute ischemic brain injuries such as anoxic encephalopathy after cardiac arrest and perinatal asphyxia (31). Randomized trials have shown that HT is also effective in improving neurological outcomes in traumatic brain injury patients (32). Neuroprotective benefits of systemic HT following ischemic stroke have been reported in clinical trials (9, 11). However, the use of HT for acute stroke treatment is still controversial and is limited by logistical challenges (9, 33).

HT initiated immediately after HI insult is neuroprotective and the degree of neuroprotection decreases linearly with the delay of initiation of cooling (34, 35). In neonatal mouse models of HI injury, HT beginning at 0 or 2 h after HI provides neuroprotection (26), while no studies have assessed the effect of HT when delayed by more than 2 h in mice. Our results showed that HT is neuroprotective up to 2 h after HI injury and the protection is lost with prolonged 4 h delay in treatment. In contrast, in a neonatal rat model, Sabir et al. (35) showed that HT delayed up to 6 h after HI insult provides neuroprotection. This may be related to differences in pathways of ischemic injury progression and neuroprotection in mice vs. rats (36). The basal metabolic rate per kg of body weight is seven times greater in mice than in humans (37) and this may play a major role in providing longer treatment windows for HT in humans in response to HI injury. Therefore, neuroprotection with 2 h delayed treatment in our protocol in mice may translate into longer time windows with HT in humans. Of relevant interest, after we reported a 2 h treatment window in neonatal mice (23), in pilot studies we documented a 6 h therapeutic window for omega-3 emulsion treatment in an adult stroke model (unpublished data). Since myelination is still occurring in the neonatal brain and the water content of the neonatal brain is greater than that of the mature brain, injury has a different appearance and time-course in the neonatal brain than in the adult brain. Cell death mechanisms have been shown to be different in the developing brain compared to that in the adult (38). The mechanisms of mitochondrial permeabilization are age-dependent and while Cyclophilin D is critical in the adult brain, B-cell lymphoma 2 (BCL-2) associated X (BAX)-related mechanisms dominate in the immature brain (39). Stroke triggers a robust inflammatory response in both adult and neonatal brain. Compared to the adult, microglial activation in neonates is much more rapid following ischemic injury. In the adult brain there is also a considerable contribution of infiltrating peripheral immune cells to the brain after stroke injury (40). In contrast, little infiltration of peripheral cells is seen acutely after neonatal stroke (41). Thus, these findings suggest differences in neonatal and adult central nervous system immune responses to injury (42, 43). We assume that these differences in ischemic injury pathophysiology and the efficacy of omega-3 fatty acids to act through these molecular pathways account for the differences in therapeutic windows observed between neonates and adults. Our present results also suggest that HT offers a very similar therapeutic window as tri-DHA treatment. A therapeutic window shorter than 6 h is recommended in neonates with HI encephalopathy (44, 45). However, few studies have demonstrated that HT initiated at 6–24 h after birth may also have benefits (46). The effective therapeutic window for HT in adult stroke patients is still not known (11, 14).

We tested whether DHA might add better neuroprotection as an adjuvant therapy to enhance the efficacy of HT after HI injury. Our results suggest that combining HT and tri-DHA does not enhance neuroprotection or extend the therapeutic window of treatment after HI injury. This is similar to recent findings from studies in newborn piglet models of HI injury, which showed that combined treatment of HT plus DHA had no additional benefits than HT alone or DHA alone treatment in reducing brain injury, oxidative stress, and inflammatory markers following HI insult (47, 48). However, another study in a neonatal rat model of HI injury reported that HT plus DHA synergistically reduced brain infarct volume and improved behavioral performances (49). Of interest, the inability to markedly enhance neuroprotection by HT plus tri-DHA treatment is not attributed to a reduction of absorption and distribution of tri-DHA emulsion particles, as demonstrated by our radiolabeled experiments. Additionally, low uptake of emulsion particles in the brain does not affect tri-DHA mediated neuroprotection in HI injury (25). Recent data from our laboratory have shown that injected tri-DHA emulsion is initially mainly taken up by the liver, which is then metabolized and secreted to plasma pools of lysophosphatidylcholine and non-esterified fatty acids, facilitating DHA brain transport (25). Further, we reported that tri-DHA administration increased DHA content in brain mitochondria and also induced a significant increase in DHA levels in blood and DHA derived specialized pro-resolving mediators (SPMs) in brain. Tri-DHA administration also increased blood levels of EPA and EPA derived SPMs in brain (24, 25). These rises in DHA, EPA, and SPMs derived from DHA and EPA might also contribute and explain the neuroprotective actions observed for DHA.

Both DHA and HT share common pathways of neuroprotection against HI injury. DHA or HT downregulate pro-apoptotic BAX and upregulate anti-apoptotic BCL-2, resulting in reduced cytochrome c release and decreased caspase activation (20, 50). DHA or HT promote activation of AKT that stimulates cell proliferation (51, 52). Further, it has been reported that in experimental stroke, DHA or HT treatment induce a decrease in microglial activation and pro-inflammatory cytokines such as interleukin 1β (IL-1β), IL-6 and tumor necrosis factor alpha (TNF-α) (53, 54). Additionally, both treatments inhibit nuclear factor kappa B (NF-κB), a transcription factor that activates many inflammatory signaling pathways (55, 56). DHA or HT have also been shown to prevent accumulation or release of excitotoxic amino acids such as glutamate (57, 58). Both DHA or HT limit reperfusion-driven acceleration in mitochondrial ROS release and protect against mitochondrial membrane permeabilization (24, 59). Thus, we speculate that overlapping neuroprotective mechanisms of DHA and HT render the combined treatment ineffective in providing enhanced neuroprotection in HI brain injury.

Previously, we reported a significant impairment in the behavioral outcomes of neonatal mice subjected to HI injury, while animals treated either with tri-DHA or neuroprotectin D1 (NPD1) had reduced infarct size with preservation of neurofunctional outcomes (24, 30). While in this study we did not measure neurofunctional outcomes following HI injury in the different groups, given the similar histological findings of HI injury and subsequent neuroprotection by HT or tri-DHA, we would predict similar levels of preservation of neurofunctional outcomes by both treatments. Furthermore, we did not delineate the potential molecular mechanisms of DHA compared to HT (10, 20). Still these limitations do not negate the significance of our work, demonstrating that post-HI tri-DHA administration provides similar degree of neuroprotection as that of HT treatment.

Currently, HT is the only established treatment for moderate to severe encephalopathy in infants (60) and is a promising strategy still under investigation for stroke therapy in adults (61). Successful clinical translation of HT for stroke requires the control of different key parameters of HT therapy including onset time, duration, depth of HT and rewarming speed (14). Although cooling a patient is simple in concept, it is a complex medical procedure that involves coordination of efforts from specially trained health care staff along with preparedness for the management issues that may arise with HT (15, 62). Using HT as a treatment for stroke usually requires settings in a tertiary care hospital and is associated with high financial costs (63). Our findings show that HT or injection of tri-DHA emulsion reduce infarct volume and the degree of neuroprotection is similar for both treatments. Omega-3 fatty acids are safe and well tolerated in humans without major adverse effects (64–66). Intravenous injections are a common feasible procedure, which can be easily performed in primary care settings. Thus, if our results using omega-3 rich lipid emulsions prove effective in treating stroke in humans, acute omega-3 therapy could be considered as an alternative cost-effective therapy for HT after ischemic organ injuries such as stroke.

## Data Availability Statement

The original contributions presented in the study are included in the article/supplementary material, further inquiries can be directed to the corresponding author/s.

## Ethics Statement

The animal study was reviewed and approved by Columbia University Institutional Animal Care and Use Committee.

## Author Contributions

DM performed all the experiments and wrote the first draft of the manuscript. HZ provided experimental assistance. RD, VT, and HZ advised on study design, on data analyses, and in revisions of the manuscript. RD, VT, HZ, and DM conceived the study, coordinated the experiments, and wrote the final version of the manuscript. All authors contributed to the article and approved the submitted version.

## Conflict of Interest

RD is a founding scientist and member of the scientific advisory board of DeckTherapeutics, Inc. (DT), a company that plans to use novel n-3 lipid emulsions to prevent tissue death after ischemic brain injury. The plans for DT do not overlap with any of the data presented in this paper. The remaining authors declare that the research was conducted in the absence of any commercial or financial relationships that could be construed as a potential conflict of interest.

## Abbreviations

DHA: docosahexaenoic acid
h: hours
HT: hypothermia
NT: normothermia
TG: triglycerides
HI: hypoxic-ischemic
SPMs: specialized pro-resolving mediators
Tri-DHA: DHA TG emulsion.
